# Methyl *rac*-(2*R*,11*S*,12*R*)-12-(2-chloro­phen­yl)-22-oxo-9,13,21-trioxapenta­cyclo­[12.8.0.0^2,11^.0^3,8^.0^15,20^]docosa-1(14),3,5,7,15(20),16,18-hepta­ene-11-carboxyl­ate

**DOI:** 10.1107/S1600536811037196

**Published:** 2011-09-17

**Authors:** K. Swaminathan, K. Sethusankar, G. Sivakumar, M. Bakthadoss

**Affiliations:** a Department of Physics, RKM Vivekananda College (Autonomous), Chennai 600 004, India; b Department of Organic Chemistry, University of Madras, Maraimalai Campus, Chennai 600 025, India

## Abstract

In the title compound C_27_H_19_ClO_6_, the coumarin ring system is not exactly planar, with a dihedral angle of 4.12 (7)° between its benzene and lactone rings. The *cis*-fused pyran rings adopt half-chair conformations. The carbometh­oxy and chloro­phenyl groups are in a *trans* configuration. The crystal packing is stabilized by inter­molecular C—H⋯O interactions, which produce a centrosymmetric *R*_2_^2^(14) dimer and two centrosymmetric *R*_2_^2^(18) dimers connecting the mol­ecules in a two-dimensional fashion.

## Related literature

For uses of coumarins, see: Kayser & Kolodziej (1997 ▶); Fan *et al.* (2001 ▶); Wang *et al.* (2002 ▶). For related structures, see: Kanchanadevi *et al.* (2011 ▶). For puckering parameters, see: Cremer & Pople (1975 ▶). For graph-set notation, see: Bernstein *et al.* (1995 ▶).
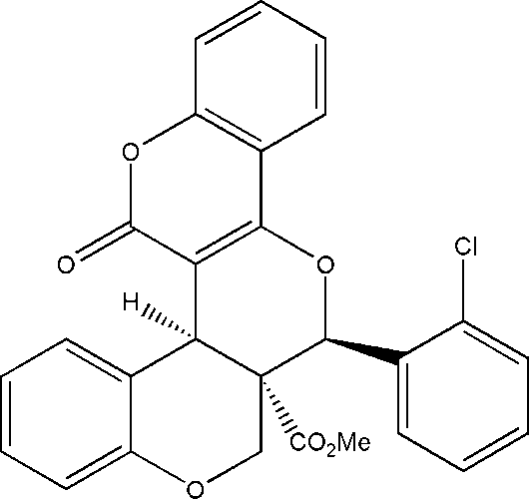


## Experimental

### 

#### Crystal data

C_27_H_19_ClO_6_
*M* *_r_* = 474.87
Triclinic, 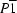

*a* = 8.4441 (3) Å
*b* = 9.7556 (3) Å
*c* = 13.8546 (5) Å
α = 73.831 (2)°
β = 82.858 (2)°
γ = 87.962 (2)°
*V* = 1087.65 (6) Å^3^
*Z* = 2
Mo *K*α radiation
μ = 0.22 mm^−1^
*T* = 293 K
0.30 × 0.25 × 0.25 mm

#### Data collection

Bruker Kappa APEXII CCD diffractometer
26685 measured reflections
6336 independent reflections
4968 reflections with *I* > 2σ(*I*)
*R*_int_ = 0.026

#### Refinement

*R*[*F* ^2^ > 2σ(*F* ^2^)] = 0.046
*wR*(*F* ^2^) = 0.143
*S* = 1.01
6336 reflections
308 parameters
H-atom parameters constrained
Δρ_max_ = 0.57 e Å^−3^
Δρ_min_ = −0.52 e Å^−3^

### 

Data collection: *APEX2* (Bruker, 2008 ▶); cell refinement: *SAINT* (Bruker, 2008 ▶); data reduction: *SAINT*; program(s) used to solve structure: *SHELXS97* (Sheldrick, 2008 ▶); program(s) used to refine structure: *SHELXL97* (Sheldrick, 2008 ▶); molecular graphics: *ORTEP-3* (Farrugia, 1997 ▶); software used to prepare material for publication: *SHELXL97* and *PLATON* (Spek, 2009 ▶).

## Supplementary Material

Crystal structure: contains datablock(s) global, I. DOI: 10.1107/S1600536811037196/ld2025sup1.cif

Structure factors: contains datablock(s) I. DOI: 10.1107/S1600536811037196/ld2025Isup2.hkl

Supplementary material file. DOI: 10.1107/S1600536811037196/ld2025Isup3.cml

Additional supplementary materials:  crystallographic information; 3D view; checkCIF report

## Figures and Tables

**Table 1:** Hydrogen-bond geometry (Å, °)

*D*—H⋯*A*	*D*—H	H⋯*A*	*D*⋯*A*	*D*—H⋯*A*
C2—H2⋯O5^i^	0.93	2.59	3.271 (2)	130
C12—H12⋯O4^ii^	0.98	2.53	3.3316 (16)	139
C23—H23⋯O5^iii^	0.93	2.47	3.355 (3)	159

Symmetry codes: (i) 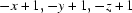; (ii) 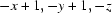; (iii) 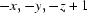.

**Fig. 1.**
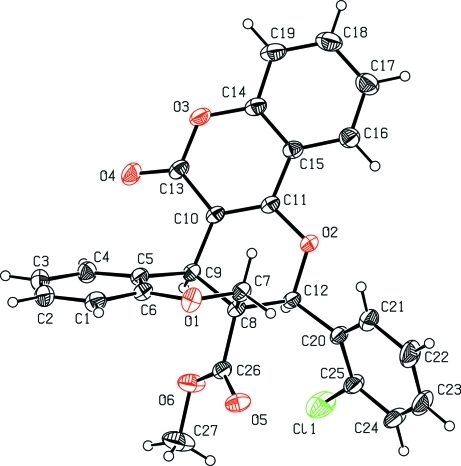
The molecular structure of the title compound with the atom numbering scheme. Displacement ellipsoids are drawn at 30% probability level. H atoms are present as small spheres of arbitary radius.

**Fig. 2.**
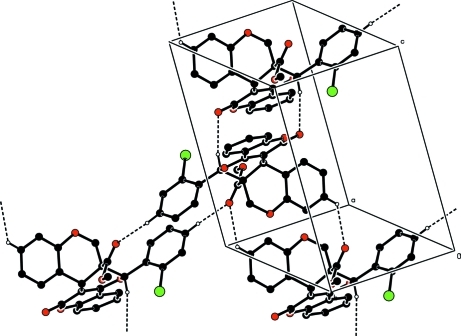
Part of the crystal structure of the title compound viewed down c axis, showing the formation of R22(14) and R22(18) dimers. C—H···O intermolecular interactions are indicated by dashed lines. Symmetry code: (i) 1 - x,1 - y,1 - z; (ii) 1 - x,1 - y,-z and (iii) -x,-y,1 - z.

## supplementary crystallographic information

### Comment

The title compound C_27_H_19_Cl O_6_ was synthesized using domino Knoevenagel intramolecular hetero-Diels-Alder reaction, used extensively in the synthesis of heterocyclic and polycyclic compounds. Coumarin derivatives find applications as active components in pesticides and additives in the manufacture of pharmaceuticals and cosmetics. They are also known to posses antibacterial (Kayser & Kolodziej, 1997), anticancer (Wang *et al.*, 2002) and steroid 5a-reductase inhibitory (Fan *et al.*, 2001) activities.

The title compound C_27_H_19_ClO_6_ comprises a chromene ring and a coumarin ring fused to alternate sides of a pyran ring. A chlorobenzene ring and a carboxylate group are also trans-attached to the same pyran ring in adjacent positions. The X-ray analysis confirms the molecular structure and atom connectivity as illustrated in Fig.1.

The chlorine atom Cl1 deviates from the least square plane of the phenyl ring (C20-C25) by 0.0568 Å and the deviation of atom O4 from the least square plane of the coumarin ring (O3,C10,C11,C13-C19) is 0.3315 (12) Å. Also, the dihedral angle between the least square planes of the pyran ring (O2,C8-C12) and the carboxylate side chain is 56.47 (6)°. The title compound exhibits sturctural similarities with a reported structure (Kanchanadevi *et al.*, 2011).

The crystal packing is stabilized by C—H..O intermolecular ineractions, which include a *R*_2_^2^(14) dimer and two *R*_2_^2^(18) dimers formed through a bifurcated hydrogen bond between a carboxylate O atom and two C atoms, one each from the nearby chromene and chlorobenzene rings, respectively (Bernstein *et al.*. 1995). (Table 1). The symmetry codes are: (i) 1 - *x*,1 - *y*,1 - *z*; (ii) 1 - *x*,1 - *y*,-*z* and (iii) -*x*,-*y*,1 - *z*. The packing arrangement of the title compound is shown in Fig.2.

### Experimental

A mixture of (*E*)-methyl 2-((2-formylphenoxy)methyl)-3-(2-chlorophenyl) acrylate (0.330 g, 1 mmol) and 4-hydroxy-2*H*-chromen-2-one (0.162 g, 1 mmol) was placed in a round bottom flask and melted at 180°C for 1 h. After completion of the reaction as indicated by TLC, the crude product was washed with 5 ml of ethylacetate:hexane mixture (1:49 ratio) which successfully provided the pure product, methyl *rac*-(2*R*,11S,12*R*)- 12-(2-chlorophenyl)-22-oxo-9,13,21-trioxapentacyclo [12.8.0.0^2,11^.0^3,8^.0^15,20^]docosa-1(14),3,5,7,15 (20),16,18- heptaene-11-carboxylate, as colourless solid in 92% yield.

### Refinement

Positions of hydrogen atoms were localized from the difference electron density maps and their distances were geometically constrained. The hydrogen atoms bound to the C atoms were treated as riding atoms, with d(C—H)=0.93 Å and *U*_iso_(H)=1.2*U*_eq_(C) for aromatic, d(C—H)=0.98 Å and *U*_iso_(H)=1.2*U*_eq_(C) for methyne, d(C—H)=0.97 Å and *U*_iso_(H)=1.2*U*_eq_(C) for methylene and d(C—H)=0.96 Å and *U*_iso_(H)=1.5*U*_eq_(C) for methyl groups. The rotation angles for methyl group were optimized by least squares.

### Crystal data

**Table:**

C_27_H_19_ClO_6_	*Z* = 2
*M**_r_* = 474.87	*F*(000) = 492
Triclinic, *P*1	*D*_x_ = 1.450 Mg m^−^^3^
Hall symbol: -P 1	Mo *K*α radiation, λ = 0.71073 Å
*a* = 8.4441 (3) Å	Cell parameters from 6336 reflections
*b* = 9.7556 (3) Å	θ = 2.2–30.0°
*c* = 13.8546 (5) Å	µ = 0.22 mm^−^^1^
α = 73.831 (2)°	*T* = 293 K
β = 82.858 (2)°	Block, colourless
γ = 87.962 (2)°	0.30 × 0.25 × 0.25 mm
*V* = 1087.65 (6) Å^3^	

### Data collection

**Table:**

Bruker Kappa APEXII CCD diffractometer	4968 reflections with *I* > 2σ(*I*)
Radiation source: fine-focus sealed tube	*R*_int_ = 0.026
graphite	θ_max_ = 30.0°, θ_min_ = 2.2°
ω scans	*h* = −11→11
26685 measured reflections	*k* = −12→13
6336 independent reflections	*l* = −19→19

### Refinement

**Table:**

Refinement on *F*^2^	Primary atom site location: structure-invariant direct methods
Least-squares matrix: full	Secondary atom site location: difference Fourier map
*R*[*F*^2^ > 2σ(*F*^2^)] = 0.046	Hydrogen site location: inferred from neighbouring sites
*wR*(*F*^2^) = 0.143	H-atom parameters constrained
*S* = 1.01	*w* = 1/[σ^2^(*F*_o_^2^) + (0.0742*P*)^2^ + 0.3227*P*] where *P* = (*F*_o_^2^ + 2*F*_c_^2^)/3
6336 reflections	(Δ/σ)_max_ < 0.001
308 parameters	Δρ_max_ = 0.57 e Å^−^^3^
0 restraints	Δρ_min_ = −0.52 e Å^−^^3^

### Special details

**Table:**

Geometry. All e.s.d.'s (except the e.s.d. in the dihedral angle between two l.s. planes) are estimated using the full covariance matrix. The cell e.s.d.'s are taken into account individually in the estimation of e.s.d.'s in distances, angles and torsion angles; correlations between e.s.d.'s in cell parameters are only used when they are defined by crystal symmetry. An approximate (isotropic) treatment of cell e.s.d.'s is used for estimating e.s.d.'s involving l.s. planes.
Refinement. Refinement of *F*^2^ against ALL reflections. The weighted *R*-factor *wR* and goodness of fit *S* are based on *F*^2^, conventional *R*-factors *R* are based on *F*, with *F* set to zero for negative *F*^2^. The threshold expression of *F*^2^ > σ(*F*^2^) is used only for calculating *R*-factors(gt) *etc*. and is not relevant to the choice of reflections for refinement. *R*-factors based on *F*^2^ are statistically about twice as large as those based on *F*, and *R*- factors based on ALL data will be even larger.

### Fractional atomic coordinates and isotropic or equivalent isotropic displacement parameters (Å^2^)

**Table:**

	*x*	*y*	*z*	*U*_iso_*/*U*_eq_	
C1	0.65779 (17)	0.56358 (17)	0.40142 (11)	0.0427 (3)	
H1	0.6784	0.5199	0.4674	0.051*	
C2	0.6817 (2)	0.70748 (18)	0.36081 (13)	0.0485 (4)	
H2	0.7199	0.7612	0.3989	0.058*	
C3	0.6491 (2)	0.77270 (17)	0.26310 (13)	0.0493 (4)	
H3	0.6674	0.8699	0.2349	0.059*	
C4	0.58945 (19)	0.69331 (15)	0.20757 (11)	0.0424 (3)	
H4	0.5632	0.7389	0.1431	0.051*	
C5	0.56753 (15)	0.54613 (14)	0.24582 (9)	0.0331 (3)	
C6	0.60257 (15)	0.48263 (14)	0.34399 (10)	0.0345 (3)	
C7	0.51941 (16)	0.25398 (14)	0.33923 (10)	0.0355 (3)	
H7A	0.4668	0.1716	0.3874	0.043*	
H7B	0.6078	0.2197	0.3004	0.043*	
C8	0.40116 (14)	0.33317 (13)	0.26765 (9)	0.0302 (2)	
C9	0.48878 (14)	0.45976 (13)	0.18856 (9)	0.0301 (2)	
H9	0.4088	0.5205	0.1515	0.036*	
C10	0.59876 (14)	0.40135 (13)	0.11382 (9)	0.0311 (2)	
C11	0.58170 (14)	0.26821 (14)	0.10487 (9)	0.0323 (2)	
C12	0.33567 (14)	0.23433 (14)	0.21156 (9)	0.0322 (2)	
H12	0.2696	0.2912	0.1614	0.039*	
C13	0.71735 (16)	0.49406 (15)	0.04264 (9)	0.0369 (3)	
C14	0.81399 (16)	0.29644 (16)	−0.01975 (10)	0.0391 (3)	
C15	0.68874 (15)	0.21021 (15)	0.03630 (9)	0.0347 (3)	
C16	0.67418 (18)	0.07365 (17)	0.02455 (11)	0.0432 (3)	
H16	0.5907	0.0146	0.0615	0.052*	
C17	0.7832 (2)	0.0264 (2)	−0.04158 (14)	0.0546 (4)	
H17	0.7722	−0.0637	−0.0505	0.066*	
C18	0.9098 (2)	0.1137 (2)	−0.09498 (14)	0.0590 (4)	
H18	0.9841	0.0806	−0.1387	0.071*	
C19	0.92701 (19)	0.2478 (2)	−0.08424 (12)	0.0531 (4)	
H19	1.0128	0.3051	−0.1195	0.064*	
C20	0.23943 (16)	0.10893 (14)	0.27860 (10)	0.0362 (3)	
C21	0.3141 (2)	−0.01302 (16)	0.33065 (13)	0.0484 (4)	
H21	0.4247	−0.0198	0.3203	0.058*	
C22	0.2274 (3)	−0.12563 (19)	0.39802 (15)	0.0646 (5)	
H22	0.2798	−0.2062	0.4331	0.078*	
C23	0.0635 (3)	−0.1172 (2)	0.41250 (16)	0.0699 (6)	
H23	0.0051	−0.1916	0.4584	0.084*	
C24	−0.0138 (2)	−0.0002 (2)	0.35981 (15)	0.0619 (5)	
H24	−0.1246	0.0045	0.3690	0.074*	
C25	0.07359 (17)	0.11175 (18)	0.29258 (12)	0.0454 (3)	
C26	0.26076 (15)	0.38165 (15)	0.33110 (10)	0.0352 (3)	
C27	0.0578 (3)	0.5498 (3)	0.33678 (18)	0.0788 (7)	
H27A	−0.0335	0.4941	0.3363	0.118*	
H27B	0.0379	0.6486	0.3049	0.118*	
H27C	0.0771	0.5384	0.4054	0.118*	
O1	0.57971 (13)	0.34145 (11)	0.39277 (7)	0.0437 (2)	
O2	0.46794 (11)	0.17495 (10)	0.15960 (7)	0.0386 (2)	
O3	0.82713 (12)	0.43425 (12)	−0.01572 (8)	0.0439 (2)	
O4	0.72725 (15)	0.62092 (12)	0.02839 (8)	0.0514 (3)	
O5	0.21355 (14)	0.31564 (13)	0.41573 (8)	0.0511 (3)	
O6	0.19620 (14)	0.50185 (13)	0.28202 (9)	0.0556 (3)	
Cl1	−0.03104 (5)	0.25730 (6)	0.22616 (5)	0.07724 (18)	

### Atomic displacement parameters (Å^2^)

**Table:**

	*U*^11^	*U*^22^	*U*^33^	*U*^12^	*U*^13^	*U*^23^
C1	0.0385 (7)	0.0535 (8)	0.0382 (7)	−0.0064 (6)	−0.0058 (5)	−0.0149 (6)
C2	0.0478 (8)	0.0524 (9)	0.0516 (8)	−0.0101 (7)	−0.0021 (6)	−0.0251 (7)
C3	0.0576 (9)	0.0389 (7)	0.0524 (9)	−0.0087 (6)	0.0015 (7)	−0.0165 (6)
C4	0.0510 (8)	0.0350 (7)	0.0385 (7)	−0.0050 (6)	−0.0011 (6)	−0.0068 (5)
C5	0.0313 (6)	0.0350 (6)	0.0319 (6)	−0.0047 (5)	−0.0005 (4)	−0.0081 (5)
C6	0.0290 (5)	0.0388 (6)	0.0342 (6)	−0.0039 (5)	−0.0029 (4)	−0.0076 (5)
C7	0.0352 (6)	0.0341 (6)	0.0331 (6)	−0.0027 (5)	−0.0057 (5)	−0.0017 (5)
C8	0.0277 (5)	0.0324 (6)	0.0271 (5)	−0.0046 (4)	−0.0006 (4)	−0.0034 (4)
C9	0.0291 (5)	0.0307 (5)	0.0274 (5)	−0.0034 (4)	−0.0031 (4)	−0.0028 (4)
C10	0.0285 (5)	0.0365 (6)	0.0255 (5)	−0.0065 (4)	−0.0017 (4)	−0.0036 (4)
C11	0.0286 (5)	0.0379 (6)	0.0276 (5)	−0.0062 (5)	−0.0005 (4)	−0.0050 (5)
C12	0.0282 (5)	0.0355 (6)	0.0307 (6)	−0.0062 (4)	0.0013 (4)	−0.0070 (5)
C13	0.0361 (6)	0.0446 (7)	0.0270 (5)	−0.0118 (5)	−0.0015 (5)	−0.0046 (5)
C14	0.0335 (6)	0.0520 (8)	0.0312 (6)	−0.0056 (5)	−0.0007 (5)	−0.0109 (5)
C15	0.0317 (6)	0.0432 (7)	0.0285 (5)	−0.0025 (5)	−0.0029 (4)	−0.0090 (5)
C16	0.0424 (7)	0.0480 (8)	0.0405 (7)	−0.0034 (6)	−0.0032 (6)	−0.0149 (6)
C17	0.0566 (9)	0.0605 (10)	0.0527 (9)	0.0029 (8)	−0.0030 (7)	−0.0275 (8)
C18	0.0516 (9)	0.0769 (12)	0.0530 (9)	0.0047 (8)	0.0062 (7)	−0.0312 (9)
C19	0.0396 (7)	0.0755 (11)	0.0428 (8)	−0.0071 (7)	0.0084 (6)	−0.0190 (8)
C20	0.0349 (6)	0.0387 (6)	0.0339 (6)	−0.0119 (5)	0.0037 (5)	−0.0100 (5)
C21	0.0486 (8)	0.0377 (7)	0.0531 (9)	−0.0091 (6)	0.0019 (7)	−0.0051 (6)
C22	0.0836 (13)	0.0398 (8)	0.0603 (10)	−0.0189 (8)	0.0015 (9)	0.0005 (7)
C23	0.0819 (13)	0.0590 (11)	0.0600 (11)	−0.0388 (10)	0.0225 (10)	−0.0105 (9)
C24	0.0466 (9)	0.0712 (12)	0.0680 (11)	−0.0298 (8)	0.0193 (8)	−0.0268 (10)
C25	0.0360 (7)	0.0533 (8)	0.0483 (8)	−0.0140 (6)	0.0025 (6)	−0.0177 (7)
C26	0.0306 (6)	0.0418 (7)	0.0335 (6)	−0.0060 (5)	−0.0007 (5)	−0.0113 (5)
C27	0.0661 (12)	0.0860 (15)	0.0753 (13)	0.0301 (11)	0.0142 (10)	−0.0209 (12)
O1	0.0515 (6)	0.0415 (5)	0.0361 (5)	−0.0080 (4)	−0.0168 (4)	−0.0013 (4)
O2	0.0364 (5)	0.0369 (5)	0.0401 (5)	−0.0104 (4)	0.0090 (4)	−0.0114 (4)
O3	0.0380 (5)	0.0518 (6)	0.0385 (5)	−0.0139 (4)	0.0087 (4)	−0.0108 (4)
O4	0.0623 (7)	0.0433 (6)	0.0423 (6)	−0.0195 (5)	0.0093 (5)	−0.0061 (4)
O5	0.0472 (6)	0.0628 (7)	0.0355 (5)	−0.0051 (5)	0.0094 (4)	−0.0065 (5)
O6	0.0474 (6)	0.0591 (7)	0.0499 (6)	0.0164 (5)	0.0078 (5)	−0.0059 (5)
Cl1	0.0375 (2)	0.0878 (4)	0.1006 (4)	−0.0003 (2)	−0.0181 (2)	−0.0121 (3)

### Geometric parameters (Å, °)

**Table:**

C1—C2	1.371 (2)	C13—O3	1.3753 (17)
C1—C6	1.3938 (19)	C14—O3	1.3700 (18)
C1—H1	0.9300	C14—C15	1.3873 (18)
C2—C3	1.383 (2)	C14—C19	1.388 (2)
C2—H2	0.9300	C15—C16	1.398 (2)
C3—C4	1.379 (2)	C16—C17	1.376 (2)
C3—H3	0.9300	C16—H16	0.9300
C4—C5	1.3955 (18)	C17—C18	1.390 (3)
C4—H4	0.9300	C17—H17	0.9300
C5—C6	1.3925 (18)	C18—C19	1.372 (3)
C5—C9	1.5247 (17)	C18—H18	0.9300
C6—O1	1.3634 (16)	C19—H19	0.9300
C7—O1	1.4195 (17)	C20—C21	1.384 (2)
C7—C8	1.5304 (17)	C20—C25	1.3900 (19)
C7—H7A	0.9700	C21—C22	1.389 (2)
C7—H7B	0.9700	C21—H21	0.9300
C8—C26	1.5279 (17)	C22—C23	1.376 (3)
C8—C9	1.5436 (16)	C22—H22	0.9300
C8—C12	1.5497 (17)	C23—C24	1.365 (3)
C9—C10	1.5203 (17)	C23—H23	0.9300
C9—H9	0.9800	C24—C25	1.387 (2)
C10—C11	1.3530 (18)	C24—H24	0.9300
C10—C13	1.4545 (16)	C25—Cl1	1.7404 (18)
C11—O2	1.3499 (14)	C26—O5	1.1959 (16)
C11—C15	1.4455 (18)	C26—O6	1.3180 (18)
C12—O2	1.4449 (15)	C27—O6	1.449 (2)
C12—C20	1.5067 (17)	C27—H27A	0.9600
C12—H12	0.9800	C27—H27B	0.9600
C13—O4	1.2021 (18)	C27—H27C	0.9600
			
C2—C1—C6	120.12 (14)	O3—C13—C10	118.33 (12)
C2—C1—H1	119.9	O3—C14—C15	120.97 (12)
C6—C1—H1	119.9	O3—C14—C19	117.76 (13)
C1—C2—C3	119.92 (14)	C15—C14—C19	121.24 (14)
C1—C2—H2	120.0	C14—C15—C16	118.92 (13)
C3—C2—H2	120.0	C14—C15—C11	117.02 (12)
C4—C3—C2	119.88 (14)	C16—C15—C11	124.05 (12)
C4—C3—H3	120.1	C17—C16—C15	120.10 (14)
C2—C3—H3	120.1	C17—C16—H16	120.0
C3—C4—C5	121.53 (14)	C15—C16—H16	120.0
C3—C4—H4	119.2	C16—C17—C18	119.80 (16)
C5—C4—H4	119.2	C16—C17—H17	120.1
C6—C5—C4	117.51 (12)	C18—C17—H17	120.1
C6—C5—C9	120.32 (11)	C19—C18—C17	121.11 (15)
C4—C5—C9	121.74 (12)	C19—C18—H18	119.4
O1—C6—C5	123.90 (12)	C17—C18—H18	119.4
O1—C6—C1	115.05 (12)	C18—C19—C14	118.78 (15)
C5—C6—C1	120.98 (12)	C18—C19—H19	120.6
O1—C7—C8	112.52 (11)	C14—C19—H19	120.6
O1—C7—H7A	109.1	C21—C20—C25	117.39 (13)
C8—C7—H7A	109.1	C21—C20—C12	120.77 (12)
O1—C7—H7B	109.1	C25—C20—C12	121.82 (13)
C8—C7—H7B	109.1	C20—C21—C22	121.45 (16)
H7A—C7—H7B	107.8	C20—C21—H21	119.3
C26—C8—C7	108.02 (10)	C22—C21—H21	119.3
C26—C8—C9	112.31 (10)	C23—C22—C21	119.55 (19)
C7—C8—C9	108.42 (10)	C23—C22—H22	120.2
C26—C8—C12	108.12 (10)	C21—C22—H22	120.2
C7—C8—C12	111.28 (10)	C24—C23—C22	120.32 (15)
C9—C8—C12	108.71 (9)	C24—C23—H23	119.8
C10—C9—C5	116.45 (10)	C22—C23—H23	119.8
C10—C9—C8	108.37 (10)	C23—C24—C25	119.77 (17)
C5—C9—C8	107.63 (9)	C23—C24—H24	120.1
C10—C9—H9	108.0	C25—C24—H24	120.1
C5—C9—H9	108.0	C24—C25—C20	121.43 (17)
C8—C9—H9	108.0	C24—C25—Cl1	117.87 (14)
C11—C10—C13	117.87 (11)	C20—C25—Cl1	120.70 (11)
C11—C10—C9	122.10 (10)	O5—C26—O6	123.95 (13)
C13—C10—C9	119.78 (11)	O5—C26—C8	123.17 (13)
O2—C11—C10	124.46 (11)	O6—C26—C8	112.87 (11)
O2—C11—C15	113.05 (11)	O6—C27—H27A	109.5
C10—C11—C15	122.47 (11)	O6—C27—H27B	109.5
O2—C12—C20	106.06 (10)	H27A—C27—H27B	109.5
O2—C12—C8	109.13 (10)	O6—C27—H27C	109.5
C20—C12—C8	115.06 (10)	H27A—C27—H27C	109.5
O2—C12—H12	108.8	H27B—C27—H27C	109.5
C20—C12—H12	108.8	C6—O1—C7	117.78 (10)
C8—C12—H12	108.8	C11—O2—C12	116.06 (10)
O4—C13—O3	115.96 (11)	C14—O3—C13	122.10 (10)
O4—C13—C10	125.69 (13)	C26—O6—C27	115.38 (14)
			
C6—C1—C2—C3	0.8 (2)	O2—C11—C15—C14	−177.14 (12)
C1—C2—C3—C4	1.3 (2)	C10—C11—C15—C14	1.25 (19)
C2—C3—C4—C5	−3.0 (2)	O2—C11—C15—C16	2.06 (19)
C3—C4—C5—C6	2.3 (2)	C10—C11—C15—C16	−179.55 (13)
C3—C4—C5—C9	174.80 (14)	C14—C15—C16—C17	0.1 (2)
C4—C5—C6—O1	176.77 (12)	C11—C15—C16—C17	−179.04 (14)
C9—C5—C6—O1	4.20 (19)	C15—C16—C17—C18	1.4 (3)
C4—C5—C6—C1	−0.15 (19)	C16—C17—C18—C19	−1.1 (3)
C9—C5—C6—C1	−172.71 (12)	C17—C18—C19—C14	−0.9 (3)
C2—C1—C6—O1	−178.59 (13)	O3—C14—C19—C18	−175.40 (15)
C2—C1—C6—C5	−1.4 (2)	C15—C14—C19—C18	2.5 (2)
O1—C7—C8—C26	−60.63 (13)	O2—C12—C20—C21	40.09 (17)
O1—C7—C8—C9	61.33 (13)	C8—C12—C20—C21	−80.64 (16)
O1—C7—C8—C12	−179.17 (10)	O2—C12—C20—C25	−141.37 (13)
C6—C5—C9—C10	−98.76 (13)	C8—C12—C20—C25	97.89 (15)
C4—C5—C9—C10	89.00 (15)	C25—C20—C21—C22	−3.0 (2)
C6—C5—C9—C8	23.08 (16)	C12—C20—C21—C22	175.57 (16)
C4—C5—C9—C8	−149.17 (12)	C20—C21—C22—C23	1.0 (3)
C26—C8—C9—C10	−166.73 (10)	C21—C22—C23—C24	1.1 (3)
C7—C8—C9—C10	73.98 (12)	C22—C23—C24—C25	−1.0 (3)
C12—C8—C9—C10	−47.12 (12)	C23—C24—C25—C20	−1.1 (3)
C26—C8—C9—C5	66.55 (13)	C23—C24—C25—Cl1	179.17 (15)
C7—C8—C9—C5	−52.74 (13)	C21—C20—C25—C24	3.1 (2)
C12—C8—C9—C5	−173.85 (10)	C12—C20—C25—C24	−175.47 (15)
C5—C9—C10—C11	138.72 (12)	C21—C20—C25—Cl1	−177.20 (12)
C8—C9—C10—C11	17.27 (16)	C12—C20—C25—Cl1	4.2 (2)
C5—C9—C10—C13	−47.09 (15)	C7—C8—C26—O5	−32.65 (17)
C8—C9—C10—C13	−168.54 (11)	C9—C8—C26—O5	−152.17 (13)
C13—C10—C11—O2	−174.11 (12)	C12—C8—C26—O5	87.89 (15)
C9—C10—C11—O2	0.2 (2)	C7—C8—C26—O6	148.50 (12)
C13—C10—C11—C15	7.69 (19)	C9—C8—C26—O6	28.98 (15)
C9—C10—C11—C15	−178.02 (11)	C12—C8—C26—O6	−90.96 (13)
C26—C8—C12—O2	−173.94 (10)	C5—C6—O1—C7	1.24 (19)
C7—C8—C12—O2	−55.46 (13)	C1—C6—O1—C7	178.32 (12)
C9—C8—C12—O2	63.88 (13)	C8—C7—O1—C6	−34.54 (16)
C26—C8—C12—C20	−54.89 (14)	C10—C11—O2—C12	15.68 (18)
C7—C8—C12—C20	63.59 (14)	C15—C11—O2—C12	−165.96 (11)
C9—C8—C12—C20	−177.08 (10)	C20—C12—O2—C11	−171.76 (10)
C11—C10—C13—O4	165.57 (14)	C8—C12—O2—C11	−47.26 (14)
C9—C10—C13—O4	−8.9 (2)	C15—C14—O3—C13	−0.6 (2)
C11—C10—C13—O3	−13.09 (18)	C19—C14—O3—C13	177.27 (13)
C9—C10—C13—O3	172.48 (11)	O4—C13—O3—C14	−168.99 (13)
O3—C14—C15—C16	175.70 (13)	C10—C13—O3—C14	9.80 (19)
C19—C14—C15—C16	−2.1 (2)	O5—C26—O6—C27	−1.6 (2)
O3—C14—C15—C11	−5.06 (19)	C8—C26—O6—C27	177.28 (16)
C19—C14—C15—C11	177.12 (13)		

### Hydrogen-bond geometry (Å, °)

**Table:**

*D*—H···*A*	*D*—H	H···*A*	*D*···*A*	*D*—H···*A*
C2—H2···O5^i^	0.93	2.59	3.271 (2)	130
C12—H12···O4^ii^	0.98	2.53	3.3316 (16)	139
C23—H23···O5^iii^	0.93	2.47	3.355 (3)	159

Symmetry codes: (i) −*x*+1, −*y*+1, −*z*+1; (ii) −*x*+1, −*y*+1, −*z*; (iii) −*x*, −*y*, −*z*+1.
